# Fibrinogen-Like Protein 1 Modulates Sorafenib Resistance in Human Hepatocellular Carcinoma Cells

**DOI:** 10.3390/ijms22105330

**Published:** 2021-05-19

**Authors:** Yeonghoon Son, Na-Rae Shin, Sung-Ho Kim, Su-Cheol Park, Hae-June Lee

**Affiliations:** 1 Division of Basic Radiation Bioscience, Korea Institute of Radiological & Medical Sciences, Seoul 01812, Korea; son-young-hun@hanmail.net (Y.S.); tlsskfo870220@gmail.com (N.-R.S.); 2 Primate Resources Center, Korea Research Institute of Bioscience and Biotechnology (KRIBB), Jeonbuk 56216, Korea; 3 College of Veterinary Medicine, Chonnam National University, Gwangju 61186, Korea; shokim@chonnam.ac.kr; 4 Department of Internal Medicine, Korea Cancer Center Hospital, Korea Institute of Radiological and Medical Sciences, Seoul 01812, Korea; hepapark@kirams.re.kr

**Keywords:** hepatocellular carcinoma, fibrinogen-like protein 1, sorafenib, resistance

## Abstract

Despite liver cancer being the second-leading cause of cancer-related death worldwide, few systemic drugs have been approved. Sorafenib, the first FDA-approved systemic drug for unresectable hepatocellular carcinoma (HCC), is limited by resistance. However, the precise mechanisms underlying this phenomenon are unknown. Since fibrinogen-like 1 (FGL1) is involved in HCC progression and upregulated after anticancer therapy, we investigated its role in regulating sorafenib resistance in HCC. FGL1 expression was assessed in six HCC cell lines (HepG2, Huh7, Hep3B, SNU387, SNU449, and SNU475) using western blotting. Correlations between FGL1 expression and sorafenib resistance were examined by cell viability, colony formation, and flow cytometry assays. FGL1 was knocked-down to confirm its effects on sorafenib resistance. FGL1 expression was higher in HepG2, Huh7, and Hep3B cells than in SNU387, SNU449, and SNU475 cells; high FGL1-expressing HCC cells showed a lower IC_50_ and higher sensitivity to sorafenib. In Huh7 and Hep3B cells, FGL1 knockdown significantly increased colony formation by 61% (*p* = 0.0013) and 99% (*p* = 0.0002), respectively, compared to that in controls and abolished sorafenib-induced suppression of colony formation, possibly by modulating ERK and autophagy signals. Our findings demonstrate that sorafenib resistance mediated by FGL1 in HCC cells, suggesting FGL1 as a potential sorafenib-resistance biomarker and target for HCC therapy.

## 1. Introduction

Liver cancer is the fourth most common cause of cancer-related deaths worldwide [1]. Primary liver cancer includes hepatocellular carcinoma (HCC) and intrahepatic cholangiocarcinoma, the former being one of the most common malignancies [2]. HCC occurs in patients with chronic liver disease and is caused by persistent liver damage, inflammation, and regeneration [3]. Since HCC is characterized by rapid progression, metastasis, and relapse, its early detection and treatment could significantly affect clinical outcomes and patient prognosis [4,5,6,7]. Treatment in the early stages of HCC includes surgical resection, liver transplantation, and topical resection [8]. However, 70% of patients who undergo resection experience tumor recurrence at 5 years [9].

Sorafenib, an oral, multikinase inhibitor, is the only FDA-approved systemic drug applicable for patients with HCC that inhibits cellular proliferation and survival-related signaling pathways [10]. Its administration has been shown to reduce the risk of death in patients with HCC [10]. The effect of sorafenib on improving survival and delaying tumor progression has been demonstrated in phase III Asia-Pacific trials conducted in South Korea, Taiwan, and China [11]. The mechanisms of action of sorafenib include induction of apoptosis of tumor cells, as well as suppression of angiogenesis by inhibiting RAS/RAF/MEK/ERK (MAPK)-mediated cell proliferation and/or vascular endothelial growth factor signaling [12]. However, the efficacy of sorafenib treatment is limited by the development of resistance [13]. Although several potential mechanisms of resistance to sorafenib, including epigenetic biological processes, transport processes, regulated cell death, and tumor microenvironment factors, have been proposed [13,14,15,16], further research is needed to clarify novel targets and mechanisms of sorafenib resistance in HCC.

Fibrinogen-like protein 1 (FGL1), also called hepassocin or hepatocyte-derived fibrinogen-related protein 1 (HFREP1), is mainly secreted from hepatocytes [17]. FGL1 shows mitotic activity in hepatocytes and is overexpressed during liver regeneration [18]. Paradoxically, FGL1 also shows a suppressive effect on the growth of hepatocellular carcinoma cells [19,20]. Its expression also has been reported that FGL1 is frequently reduced or absent in human HCC tissue [21], and in vivo disruption of FGL1 accelerates HCC development [18]. A recent study reported that FGL1 is a new major ligand of lymphocyte-activation gene 3 (LAG-3), which is an immune inhibitory receptor. Moreover, the potential of FGL1 in cancer immunotherapy was recently suggested [22]. A previous study attempted to identify potential therapeutic target genes for HCC by using Gene Expression Omnibus (GEO) and The Cancer Genome Atlas (TCGA) databases [23], and FGL1 was identified as a possible biomarker in lung adenocarcinoma [24]. However, little is known about the impact of FGL1 on sorafenib resistance or the relationship between changes in the FGL1 level and HCC progression.

The present study aimed to explore basal FGL1 expression in six HCC cell lines (Hep3B, Huh7, HepG2, SNU387, SNU449, and SNU475) and investigate its relationship with susceptibility to sorafenib treatment. We examined changes in proliferation, viability, and resistance-related molecular signaling in response to sorafenib treatment in vitro.

## 2. Results

### 2.1. Basal FGL1 Levels in HCC Cell Lines Are Associated with Cell Viability in Response to Sorafenib Treatment

**Figure 1:**
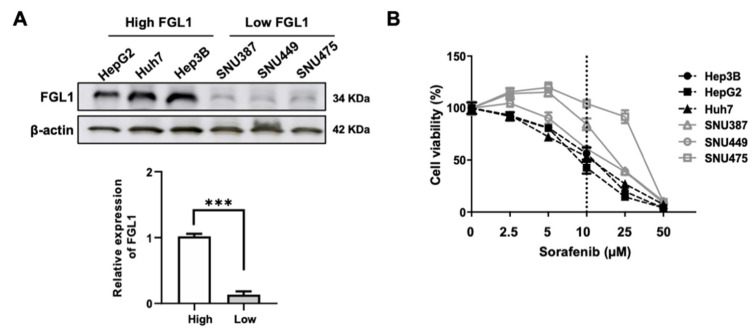
FGL1 expression level and viability in HepG2, Huh7, Hep3B, SNU387, SNU449, and SNU475 hepatocellular carcinoma (HCC) cell lines. (**A**) Expression of FGL1 in the six HCC cell lines, as determined through immunoblotting. Data are presented as the mean ± standard deviation (SD) (n = 3). *** *p* < 0.001, significantly different from HCC cells with high FGL1-expressing. (**B**) Cell viability in the six HCC cell lines after sorafenib treatment. Data are presented as the mean ± standard deviation (n = 6).

Basal FGL1 levels in the six HCC cell lines were investigated using western blotting. The results showed obvious FGL1 expression in HCC cell lines, with expression levels being notably higher in HepG2, Huh7, and Hep3B cells than in SNU387, SNU449, and SNU475 cells (Figure 1A). To examine the viability of each HCC cell line after sorafenib treatment, we treated all six cell lines with various concentrations of sorafenib. The three HCC cell lines with high FGL1 expression were more sensitive to sorafenib than those with low FGL1 expression (Figure 1B). IC_50_ (half-maximum inhibitory concentration) values of sorafenib in high-FGL1 expressing HCC cells (HepG2, Huh7, and Hep3B) were 2–3 times lower than those in low-FGL1 HCC cells (Table 1).

### 2.2. Sensitivity to Sorafenib Is Correlated with Endogenous FGL1 Levels in HCC Cell Lines

**Figure 2:**
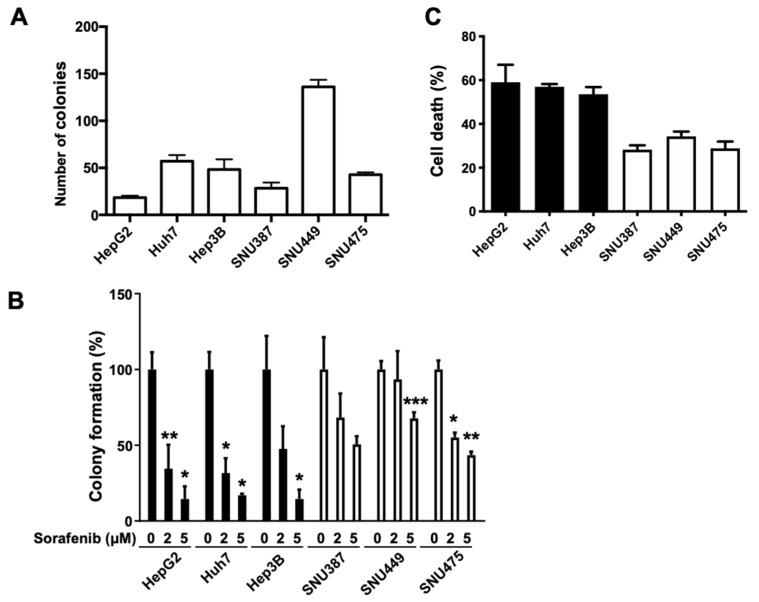
Effect of sorafenib on the cell colony forming capacity and cell death of HepG2, Huh7, Hep3B, SNU387, SNU449, and SNU475 hepatocellular carcinoma (HCC) cell lines. (**A**) Number of colonies after seeding 200 cells of each HCC cell line in the plate. (**B**) Effect of sorafenib on the colony forming potential of HCC cells. The six HCC cell lines were incubated for 7 days in the presence of 0, 2, or 5 µM sorafenib. Data are presented as the mean ± standard deviation (SD) (n = 3). * *p* < 0.05, ** *p* < 0.01, and *** *p* < 0.001, significantly different from 0 µM sorafenib-treated cells. (**C**): Effect of sorafenib on cell death measured by flow cytometry using Annexin V-FITC and propidium iodide (PI) staining. The graph presents mean ± SD values of three independent experiments.

To investigate the effect of sorafenib on the growth of HCC cells, we employed the colony forming assay. We treated the six HCC cell lines with 0, 2, and 5 μM sorafenib and counted the number of colonies that developed. Results showed that the number of colonies was different across HCC cell lines (Figure 2A). However, after sorafenib treatment, a dose-dependent decrease in colony number was observed in all six HCC cell lines; colonies of high-FGL1 expressing HCC cells were fewer than those of low-FGL1 expressing cells (Figure 2B). The results indicated that cell death due to sorafenib treatment in high-FGL1 expressing HCC cells (HepG2, Huh7, and Hep3B) was 2–3 times higher than that in low-FGL1 cells (Figure 2C).

### 2.3. Effects of Sorafenib on MAPK and Autophagy Pathways Differ Based on the FGL1 Expression Level

**Figure 3:**
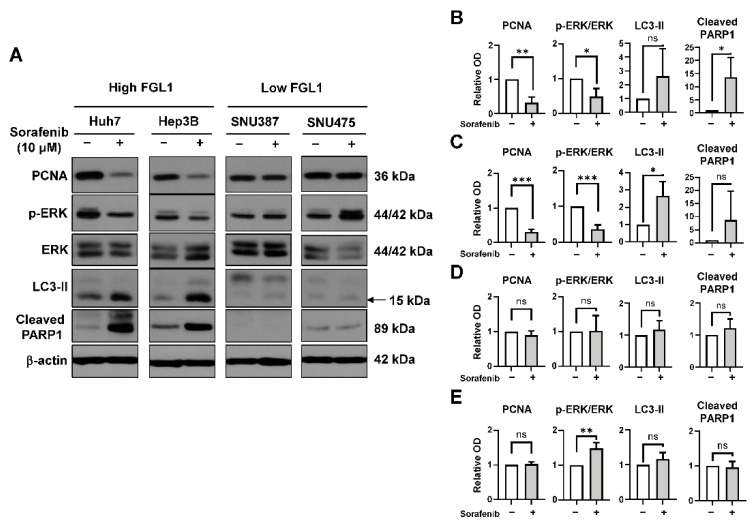
Effect of sorafenib on cell death and cell proliferation factors in high FGL1- and low FGL1-expressing hepatocellular carcinoma (HCC) cell lines. (**A**): Protein expression was analyzed by western blotting 48 h after treatment with 10 μM sorafenib. Expression levels of PCNA, p-ERK/ERK, LC3-II, and cleaved PARP1 quantified in Huh7 (**B**), Hep3B (**C**), SNU387 (**D**), and SNU475 (**E**) cell lines. Data are presented as the mean ± standard deviation (SD) (n = 3). * *p* < 0.05, ** *p* < 0.01, and *** *p* < 0.001, significantly different from 0 µM sorafenib-treated cells.

To examine whether the potential mechanism underlying the therapeutic action of sorafenib differed depending on the basal level of FGL1 in HCC cell lines, we measured the levels of proteins related to cell proliferation, autophagy, and apoptosis by western blotting (Figure 3A). Sorafenib treatment in Huh7 and Hep3B cells lowered the levels of PCNA and phosphorylated ERK while increasing those of LC3-II and cleaved PARP1 (Figure 3B,C). However, in SNU387 and SNU475 cells, sorafenib affected neither PCNA and phosphorylated ERK nor LC3-II, and cleaved PARP1 (Figure 3D,E).

### 2.4. Suppression of FGL1 Mediates Colony Formation and Death of HCC Cells after Sorafenib Treatment

**Figure 4:**
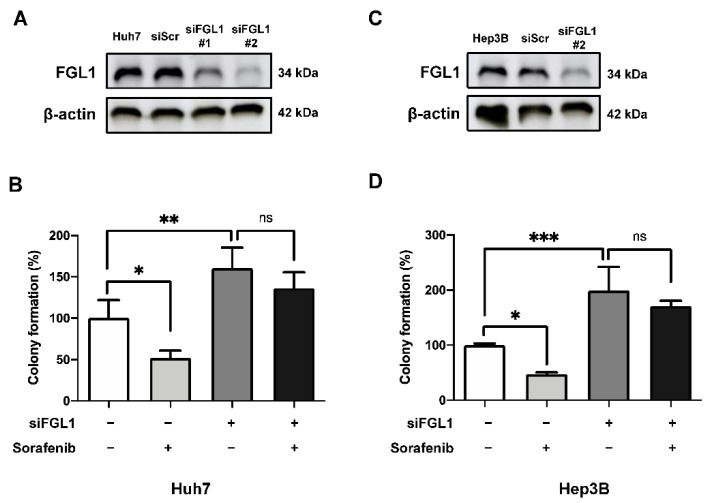
Effect of silencing FGL1 in Huh7 and Hep3B cells on their sorafenib sensitivity. (**A**) Expression of FGL1 in Huh7 cells 24 h after FGL1 siRNA transfection. (**B**) Colony formation of Huh7 cells following siFGL1 and/or sorafenib (2 µM) treatment. (**C**) Expression of FGL1 in Hep3B cells 24 h after FGL1 siRNA transfection. (**D**) Colony formation of Hep3B cells following siFGL1 and/or sorafenib (2 µM) treatment. Graphs present mean ± standard deviation values (n = 4). * *p* < 0.05, ** *p* < 0.01, and *** *p* < 0.001, significantly different from scrambled siRNA-treated cells. siFGL1 (−), treated with siScr; Sorafenib (−), treated with vehicle (DMSO); siScr, scrambled siRNA; siFGL1, FGL1 siRNA.

To investigate the impact of FGL1 on HCC cell lines, we knocked down endogenous FGL1 in two high FGL1-expressing HCC cell lines, Huh7 and Hep3B, using siRNA. FGL1 levels were decreased in both cell lines after siFGL1 transfection (Figure 4A,C). Knockdown of FGL1 increased the colony formation ability of Huh7 and Hep3B cells by 61% (*p* = 0.0013) and 99% (*p* = 0.0002), respectively (Figure 4B,D). The anticancer effect of sorafenib was notably interrupted by siFGL1 in Huh7 (−24%) and Hep3B (−28%) cells, in contrast to the anticancer effect of sorafenib mediated by scrambled siRNA in Hur7 (−48%) and Hep3B (−52%) (Figure 4B,D).

**Figure 5:**
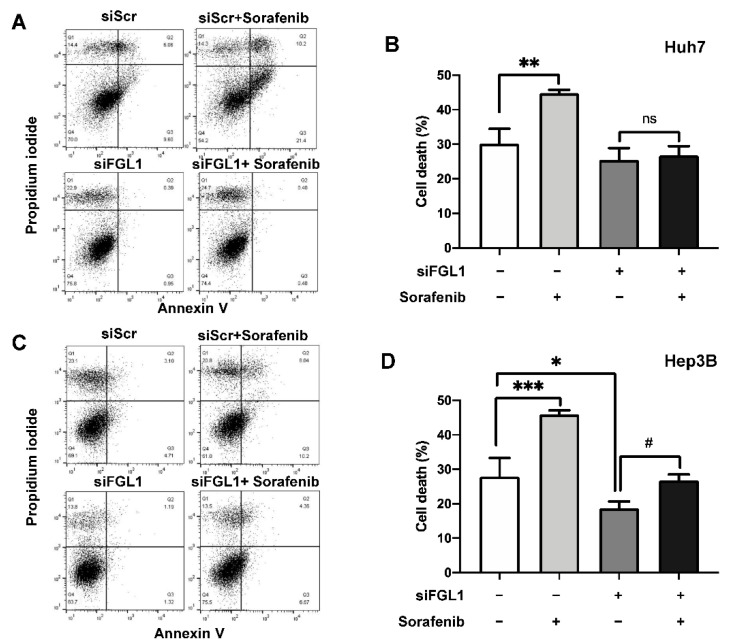
Effect of FGL1 knockdown on sorafenib-induced cell death in Huh7 and Hep3B cell lines. Flow cytometry results of Huh7 (**A**,**B**) and Hep3B (**C**,**D**) cells are shown. The cell death percentage was estimated using the sum of the upper left, upper right, and lower right quadrants in each dot plot. The graphs present percentage of cell death. All values are presented as the mean ± standard deviation of three independent experiments. * *p* < 0.05, ** *p* < 0.01, and *** *p* < 0.001, significantly different from scrambled siRNA-treated cells; # *p* < 0.05, significantly different from FGL1 siRNA-treated cells. siFGL1 (−), treated with siScr; Sorafenib (−), treated with vehicle (DMSO); siScr, scrambled siRNA; siFGL1, FGL1 siRNA.

To further investigate the effect of siFGL1 on resistance to sorafenib, cell death was examined by flow cytometry of siFGL1- and/or sorafenib-treated HCC cells (Figure 5A,C). Flow cytometric analysis revealed that sorafenib treatment significantly increased the cell death rate for both Huh7 and Hep3B cells (Figure 5). However, knockdown of FGL1 impeded sorafenib-induced cell death for both Huh7 and Hep3B cells (Figure 5B,D), although there were slight increases in the sorafenib-induced cell death rate in Hep3B cells with siFGL1.

### 2.5. Knockdown of FGL1 Modulates Sorafenib-Induced p-ERK and Autophagy Signaling

**Figure 6:**
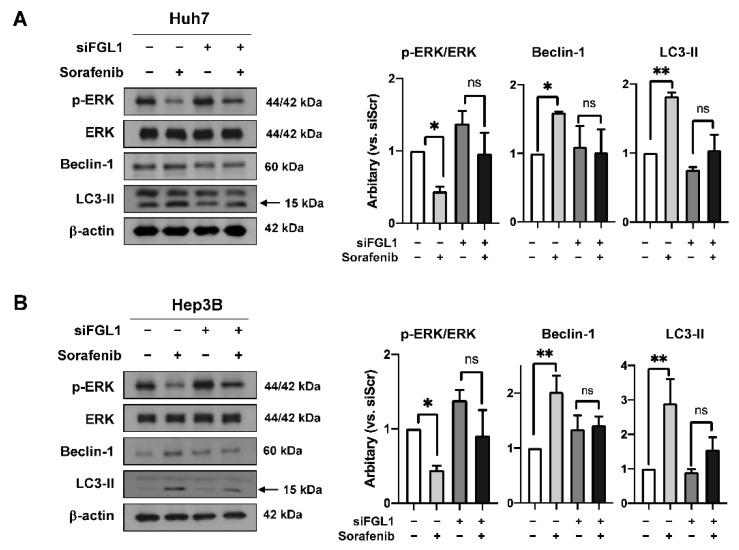
Effect of FGL1 knockdown on p-ERK, ERK, Beclin-1, and LC3-II in hepatocellular carcinoma (HCC) cells following sorafenib treatment. Representative western blot and data from Huh7 (**A**) and Hep3B (**B**) cells. Data are presented as the mean ± standard deviation (n = 3). * *p* < 0.05 and ** *p* < 0.01, compared to siScr-treated cells. siFGL1 (−), treated with siScr. Sorafenib (−), treated with vehicle (DMSO).

As shown previously, sorafenib inhibited the phosphorylation of ERK and facilitated autophagic signaling in high FGL1-expressing HCC cells (Figure 3 and Figure 6). We analyzed p-ERK, Beclin-1, and LC3-II after sorafenib treatment in HCC cells with FGL1 knockdown. Consistent with flow cytometry and colony formation assay results, sorafenib treatment significantly suppressed the phosphorylation of ERK in both two cell lines (*p* = 0.016 in Huh7 and *p* = 0.028 in Hep3B), whereas siFGL1 abolished those effects on p-ERK. Moreover, sorafenib-induced activation of autophagy signals, represented by Beclin-1 and LC3-II, was alleviated by siFGL1 (Figure 6).

## 3. Discussion

Sorafenib is an FDA-approved therapeutic agent for advanced HCC [25]. However, resistance to systemic sorafenib therapy is becoming increasingly common. To improve the antitumor effect of sorafenib, its potential mechanism and therapeutic targets should be clarified. In this study, we investigated FGL1 as a promising biomarker that predicts therapeutic response to sorafenib in HCC. We found that HCC cell lines showed different basal expression levels of FGL1, and there was an association between endogenous expression levels of FGL1 and sensitivity to the sorafenib-induced anticancer effect. In high FGL1-expressing HCC cells, knockdown of FGL1 mediated resistance to sorafenib, indicated by a higher proliferation rate and colony forming ability and lower cell death, via the phosphorylation of ERK and autophagy signaling.

FGL1, secreted by parenchymal hepatocytes, is upregulated during liver regeneration and is involved in liver cell growth [26]. A previous study reported that FGL1 expression is elevated in HCC tissue relative to that in adjacent normal liver tissues [22]. In addition, high plasma FGL1 expression is associated with poor response to immunotherapy, suggesting FGL1 as a potential biomarker for predicting the outcome of cancer immunotherapy [17]. Moreover, FGL1 expression had been reported to be upregulated both in vivo and in vitro following irradiation, indicating the possibility of FGL1 as a biomarker for liver injury [27]. In contrast, overall survival time was found to be remarkably shorter in patients with gastric cancer with high FGL1 expression than in gastric cancer patients showing low FGL1 expression [28]. Thus, controversy remains regarding the effects of FGL1 on cancer cell progression, thereby calling for further investigation. In the present study, we first examined the basal FGL1 expression levels in six HCC cell lines, HepG2, Huh7, Hep3B, SNU387, SNU449, and SNU475 (Figure 1). We found that HCC cells with high levels of FGL1 showed higher sensitivity to sorafenib than those with low levels of FGL1 (Figure 2). These results indicated that sensitivity to sorafenib treatment is possibly related to the basal levels of FGL1 in HCC cell lines.

The MAPK signaling cascade has been reported to be essential for cellular communication, involving cell growth, survival, and differentiation [29]. Thus, considerable effort has been invested in deciphering the molecular mechanisms involved in this pathway for the development of cancer therapies [30]. Sorafenib is a potent multikinase inhibitor capable of facilitating apoptosis, mitigating angiogenesis, and suppressing tumor cell proliferation [31] in HCC [32] and prostate cancer cells [33] through MAPK [12]. It was previously reported that the regulation of cell death, especially autophagy and ferroptosis, is involved in sorafenib resistance in HCC. It has also been reported that c-Jun expression is associated with sorafenib resistance [34]; however, the precise mechanisms associated with sorafenib resistance in HCC cell lines remain largely unknown, and requires further investigation. In the present study, HCC cells with high expression levels of FGL1, namely Huh7 and Hep3B, showed induction of autophagy and apoptosis-related signals and a reduction in ERK phosphorylation following sorafenib treatment. However, these changes were not found in HCC cells expressing low levels of FGL1, namely SNU387 and SNU475 (Figure 3). These findings indicated that sorafenib sensitivity is possibly related to the activation of ERK and autophagy signaling pathways in HCC cells; hence, measurement of basal FGL1 expression levels could be useful as a predictor of sorafenib sensitivity.

To investigate the role of FGL1 in the response to sorafenib in HCC cell lines, we modulated FGL1 expression levels in high FGL1-expressing HCC cells, Huh7 and Hep3B, via FGL1 siRNA. Notably, siFGL1 treatment in HCC cells resulted in lower cell death and higher colony forming activity compared to those with scrambled siRNA. Whereas sorafenib treatment significantly increased cell death and reduced colony formation in both Huh7 and Hep3B cell lines (Figure 2), knockdown of FGL1 alleviated sorafenib-induced anticancer effects, as represented by the levels of cell death and colony formation (Figure 4 and Figure 5). Moreover, we found that siFGL1-induced resistance to sorafenib in HCC was associated with a reduction in p-ERK and autophagy signaling (Beclin-1/LC3-II) by western blotting (Figure 6). Previously, Zhang et al. [32] suggested that the phosphorylation status of ERK is a potential predictor of sorafenib sensitivity in HCC; basal p-ERK levels increase in accordance with their metastatic potential. Furthermore, studies have indicated a critical role for autophagy in sorafenib resistance in HCC, as measured by changes in the activity of IRE1, Akt, mTORC1, and others. Moreover, Tai et al. [35] reported that sorafenib activates autophagy through the release of Beclin-1 binding to Mcl-1. Taken together, our results suggested that FGL1 is involved in resistance to sorafenib in HCC via p-ERK/autophagy signaling. However, additional studies using multiple cell lines to assess the potential regulatory mechanism in HCC cell lines with different origins are required to determine the possible role of FGL1 expression in drug resistance.

There have been controversial results with respect to FGL1 expression levels and their impact on cancer therapy. Our results showed that siFGL1 in high FGL1-expressing cells increased colony formation; these results are consistent with previous reports showing that FGL1 exerts an inhibitory effect on HCC growth and functions as a tumor suppressor in HCC proliferation [21] and that knockdown of FGL1 accelerates HCC development in vivo [18]. Further, Bie et al. reported, via through TCGA and GEO database data mining and in vitro functional experiments, that the loss of FGL1 promotes cell growth, epithelial-mesenchymal transition (EMT), and angiogenesis in LKB1-mutant lung adenocarcinoma [24]. However, Sun et al. reported that FGL1 confers gefitinib resistance and that the knockdown of FGL1 in lung adenocarcinoma cells induces antitumor effects in vivo [36]. Therefore, further preclinical and clinical studies of HCC are necessary to clarify the role of FGL1 and its impact on HCC.

There are limitations to this study. First, we demonstrated the effect of FGL1 suppression on sorafenib resistance using high FGL1-expressing HCC cell lines, but did not overexpress FGL1 in low FGL1-expressing cells. Further studies to clarify the impact of FGL1 overexpression on HCC are needed. Second, additional clinical evidence is needed to confirm our conclusion.

The current study demonstrated that the effects of sorafenib treatment on cell viability and colony formation are significantly correlated with the basal FGL1 level in HCC cell lines. Knockdown of FGL1 decreased sorafenib-induced apoptosis and suppression of cell proliferation in HCC cell lines with high FGL1 expression, probably through ERK/autophagy signaling. Our results collectively suggest that FGL1 is a potential biomarker for sorafenib resistance in HCC and a promising target for HCC therapy.

## 4. Materials and Methods

### 4.1. Cell Culture

The cell lines and their culture media were as follows: HepG2 (ATCC, Manassas, VA, USA), DMEM; Huh7 (KCLB, Korean Cell Line Bank, Seoul, Korea), RPMI1640; Hep3B (KCLB), DMEM; SNU387 (KCLB), RPMI1640; SNU475 (KCLB), RPMI1640; SNU 449 (KCLB), RPMI1640. The medium for each cell line was supplemented with 10% FBS and 1% antibiotic and cultured in an incubator at 37 °C and 5% CO_2_.

### 4.2. Reagents and Treatment

Sorafenib purchased from Selleckchem Inc (Houston, TX, USA) was dissolved in dimethyl sulfoxide (DMSO). DMSO was used as a vehicle. For FGL1 silencing in HCC cells, scrambled siRNA (siScr) and FGL1 siRNA (siFGL1) were purchased from IDT^®^ (Integrated DNA Technologies, IA, USA). siRNAs were transfected using the Lipofectamine 2000 (Invitrogen, San Diego, CA, USA) following manufacturer’s instructions. siRNA sequences were as follows: 5′-GGGACAGAGAUCAUGACAACUAUGA-3′ for siFGL1 (#2) and 5′-CGUUAAUCGCGUAUAAUACGCGUAT-3′ for siScr.

### 4.3. Cell Viability Assay

The cell viability assay was performed using 3-(4,5-dimethylthiazol-2-yl)-2,5-diphenyltetrazolium bromide (MTT). For this, 3 × 10^4^ cells were seeded in 24-well plates and treated with different concentrations of sorafenib (2.5, 5, 10, 25, and 50 μM). After 48 h, MTT solution was added (final concentration 0.5 mg/mL) to each well and then incubated for 2 h at 37 °C. Next, MTT solution was removed and DMSO was added to each well. Optical density was measured at 570 nm using a microplate reader (Molecular Devices Ins, Sunnyvale, CA, USA).

### 4.4. Colony Forming Assay

To assess the effectiveness of sorafenib on six different HCC cell lines, we conducted a colony forming assay. From each cell line, 300 cells were plated in a 60-mm dish. After 24 h, sorafenib was added at 0, 2, or 5 μM, and the cells were incubated at 37 °C with 5% CO_2_ for 7~10 days. The cells were washed with PBS twice and fixed with 10% neutral buffered formalin in PBS solution. Then, the cells were stained with 0.5% crystal violet for 30 min, washed with running water, and the number of colonies was counted. A colony was defined as consisting of more than 50 cells.

### 4.5. Western Blotting

Immunoblotting was performed as described previously [27]. Briefly, cells were lysed with RIPA buffer supplemented with protease inhibitor. The protein concentration was then determined using Bradford reagent (Bio-Rad, Hercules, CA, USA) according to the manufacturer’s instructions. Equal amounts of protein (15–40 μg) were separated on 10% SDS-PAGE gel and transferred onto a nitrocellulose membrane at 100 V for 90 min. The membrane was incubated in 5% skim milk with PBS containing tween 20 (PBST) for at least 1 h to block non-specific binding. Next, the membrane was incubated with the primary antibody at 4 °C overnight. The following primary antibodies were used to detect target proteins: anti-FGL1 (1:1000 dilution; Proteintech, Rosemont, IL, USA), anti-PCNA (1:1000 dilution; Santa Cruz Biotechnology, Dallas, TX, USA), anti-phosphorylated ERK (1:1000 dilution; Cell Signaling Technologies, Danvers, MA, USA), anti-ERK (1:1000 dilution; Cell Signaling Technologies), anti-LC3-II (1:3000 dilution; Novus Biologicals, Littleton, CO, USA), anti-cleaved PARP1 (1:1000 dilution; Cell Signaling Technologies), anti-Beclin-1 (1:1000 dilution; Cell Signaling Technologies), and anti-β-actin (1:2000 dilution; Sigma-Aldrich, St. Louis, MO, USA). The membrane was thereafter incubated with the appropriate horseradish peroxidase-conjugated secondary antibody (1:3000 dilution; Santa Cruz Biotechnology) for 1 h at room temperature. The membrane was finally washed with PBST and then developed using an enhanced chemiluminescence kit (Perkin Elmer, Waltham, MA, USA). Protein bands were quantified using ImageJ software (National Institutes of Health, Bethesda MD, USA) and corrected by subtracting the measured intensity from that of β-actin.

### 4.6. Annexin V/Propidium Iodide Staining

The Annexin V-FITC Apoptosis Detection Kit (556547, BD Biosciences, San Jose, CA, USA) was used to detect apoptotic cell death. Cells were seeded (1 × 10^4^ per well) into 60 mm dishes and incubated with sorafenib (10 μM). After 48 h, cells were washed once in cold PBS and resuspended in 500 µL of 1× binding buffer containing 5 µL of annexin-FITC and 5 µL of propidium iodide. Then, the apoptotic cells were analyzed immediately using the FACSCalibur™ flow cytometer (Becton-Dickinson, Franklin Lakes, NJ, USA).

### 4.7. Statistical Analysis

Data are represented as the mean ± standard deviation. Statistical significance was determined using one-way analysis of variance followed by Tukey’s multiple comparison test using GraphPad Prism (version 8.4.3).

## Figures and Tables

**Table 1:** IC_50_ of sorafenib against HCC cell lines.

Cell Line	Basal FGL1 Level	IC_50_ (µM)
Hep3B	High	12.56 ± 0.79
HepG2	High	10.14 ± 0.92
Huh7	High	11.55 ± 1.01
SNU387	Low	20.77 ± 0.96
SNU449	Low	17.67 ± 0.83
SNU475	Low	38.32 ± 1.54

Values of IC_50_ are the mean ± SD.

## Data Availability

The data that support the findings of this study are available from the corresponding author upon reasonable request.

## Abbreviations

DMSO	dimethyl sulfoxide
FGL1	fibrinogen-like 1
HCC	hepatocellular carcinoma
HFREP1	hepatocyte-derived fibrinogen-related protein 1
MTT	3-(4,5-dimethylthiazol-2-yl)-2,5-diphenyltetrazolium bromide
PBST	PBS containing tween 20
SD	standard deviation
siFGL1	FGL1 siRNA
siScr	scrambled siRNA

## References

[1] Sung H., Ferlay J., Siegel R.L., Laversanne M., Soerjomataram I., Jemal A., Bray F. (2021). Global cancer statistics 2020: GLOBOCAN estimates of incidence and mortality worldwide for 36 cancers in 185 countries. CA Cancer J. Clin..

[2] Massarweh N.N., El-Serag H.B. (2017). Epidemiology of Hepatocellular Carcinoma and Intrahepatic Cholangiocarcinoma. Cancer Control.

[3] Yu L.X., Ling Y., Wang H.Y. (2018). Role of nonresolving inflammation in hepatocellular carcinoma development and progression. NPJ Precis. Oncol..

[4] Tang Z.Y. (2001). Hepatocellular carcinoma—Cause, treatment and metastasis. World J. Gastroenterol..

[5] Fujiwara N., Friedman S.L., Goossens N., Hoshida Y. (2018). Risk factors and prevention of hepatocellular carcinoma in the era of precision medicine. J. Hepatol..

[6] Cassim S., Raymond V.A., Dehbidi-Assadzadeh L., Lapierre P., Bilodeau M. (2018). Metabolic reprogramming enables hepatocarcinoma cells to efficiently adapt and survive to a nutrient-restricted microenvironment. Cell Cycle.

[7] Cassim S., Raymond V.A., Lacoste B., Lapierre P., Bilodeau M. (2018). Metabolite profiling identifies a signature of tumorigenicity in hepatocellular carcinoma. Oncotarget.

[8] Belghiti J., Kianmanesh R. (2005). Surgical treatment of hepatocellular carcinoma. HPB (Oxford).

[9] Duffy J.P., Vardanian A., Benjamin E., Watson M., Farmer D.G., Ghobrial R.M., Lipshutz G., Yersiz H., Lu D.S., Lassman C. (2007). Liver transplantation criteria for hepatocellular carcinoma should be expanded: A 22-year experience with 467 patients at UCLA. Ann. Surg..

[10] Ziogas I.A., Tsoulfas G. (2017). Evolving role of Sorafenib in the management of hepatocellular carcinoma. World J. Clin. Oncol..

[11] Raoul J.L., Frenel J.S., Raimbourg J., Gilabert M. (2019). Current options and future possibilities for the systemic treatment of hepatocellular carcinoma. Hepat. Oncol..

[12] Wilhelm S.M., Adnane L., Newell P., Villanueva A., Llovet J.M., Lynch M. (2008). Preclinical overview of sorafenib, a multikinase inhibitor that targets both Raf and VEGF and PDGF receptor tyrosine kinase signaling. Mol. Cancer Ther..

[13] Mendez-Blanco C., Fondevila F., Garcia-Palomo A., Gonzalez-Gallego J., Mauriz J.L. (2018). Sorafenib resistance in hepatocarcinoma: Role of hypoxia-inducible factors. Exp. Mol. Med..

[14] Huang D., Yuan W., Li H., Li S., Chen Z., Yang H. (2018). Identification of key pathways and biomarkers in sorafenib-resistant hepatocellular carcinoma using bioinformatics analysis. Exp. Ther. Med..

[15] Chen J., Jin R., Zhao J., Liu J., Ying H., Yan H., Zhou S., Liang Y., Huang D., Liang X. (2015). Potential molecular, cellular and microenvironmental mechanism of sorafenib resistance in hepatocellular carcinoma. Cancer Lett..

[16] Tang W., Chen Z., Zhang W., Cheng Y., Zhang B., Wu F., Wang Q., Wang S., Rong D., Reiter F.P. (2020). The mechanisms of sorafenib resistance in hepatocellular carcinoma: Theoretical basis and therapeutic aspects. Signal Transduct. Target Ther..

[17] Wang J., Sanmamed M.F., Datar I., Su T.T., Ji L., Sun J., Chen L., Chen Y., Zhu G., Yin W. (2019). Fibrinogen-like Protein 1 Is a Major Immune Inhibitory Ligand of LAG-3. Cell.

[18] Nayeb-Hashemi H., Desai A., Demchev V., Bronson R.T., Hornick J.L., Cohen D.E., Ukomadu C. (2015). Targeted disruption of fibrinogen like protein-1 accelerates hepatocellular carcinoma development. Biochem. Biophys. Res. Commun..

[19] Cao M.M., Xu W.X., Li C.Y., Cao C.Z., Wang Z.D., Yao J.W., Yu M., Zhan Y.Q., Wang X.H., Tang L.J. (2011). Hepassocin regulates cell proliferation of the human hepatic cells L02 and hepatocarcinoma cells through different mechanisms. J. Cell Biochem..

[20] Yan J., Yu Y., Wang N., Chang Y., Ying H., Liu W., He J., Li S., Jiang W., Li Y. (2004). LFIRE-1/HFREP-1, a liver-specific gene, is frequently downregulated and has growth suppressor activity in hepatocellular carcinoma. Oncogene.

[21] Yu H.T., Yu M., Li C.Y., Zhan Y.Q., Xu W.X., Li Y.H., Li W., Wang Z.D., Ge C.H., Yang X.M. (2009). Specific expression and regulation of hepassocin in the liver and down-regulation of the correlation of HNF1alpha with decreased levels of hepassocin in human hepatocellular carcinoma. J. Biol. Chem..

[22] Guo M., Yuan F., Qi F., Sun J., Rao Q., Zhao Z., Huang P., Fang T., Yang B., Xia J. (2020). Expression and clinical significance of LAG-3, FGL1, PD-L1 and CD8(+)T cells in hepatocellular carcinoma using multiplex quantitative analysis. J. Transl. Med..

[23] Wei Z., Liu Y., Qiao S., Li X., Li Q., Zhao J., Hu J., Wei Z., Shan A., Sun X. (2019). Identification of the potential therapeutic target gene UBE2C in human hepatocellular carcinoma: An investigation based on GEO and TCGA databases. Oncol. Lett..

[24] Bie F., Wang G., Qu X., Wang Y., Huang C., Wang Y., Du J. (2019). Loss of FGL1 induces epithelialmesenchymal transition and angiogenesis in LKB1 mutant lung adenocarcinoma. Int. J. Oncol..

[25] Furuse J. (2008). Sorafenib for the treatment of unresectable hepatocellular carcinoma. Biologics.

[26] Hara H., Uchida S., Yoshimura H., Aoki M., Toyoda Y., Sakai Y., Morimoto S., Fukamachi H., Shiokawa K., Hanada K. (2000). Isolation and characterization of a novel liver-specific gene, hepassocin, upregulated during liver regeneration. Biochim. Biophys. Acta.

[27] Han N.K., Jung M.G., Jeong Y.J., Son Y., Han S.C., Park S., Lim Y.B., Lee Y.J., Kim S.H., Park S.C. (2019). Plasma Fibrinogen-Like 1 as a Potential Biomarker for Radiation-Induced Liver Injury. Cells.

[28] Zhang Y., Qiao H.X., Zhou Y.T., Hong L., Chen J.H. (2018). Fibrinogenlikeprotein 1 promotes the invasion and metastasis of gastric cancer and is associated with poor prognosis. Mol. Med. Rep..

[29] Cargnello M., Roux P.P. (2011). Activation and function of the MAPKs and their substrates, the MAPK-activated protein kinases. Microbiol. Mol. Biol. Rev..

[30] Degirmenci U., Wang M., Hu J. (2020). Targeting Aberrant RAS/RAF/MEK/ERK Signaling for Cancer Therapy. Cells.

[31] Shi Y.H., Ding Z.B., Zhou J., Hui B., Shi G.M., Ke A.W., Wang X.Y., Dai Z., Peng Y.F., Gu C.Y. (2011). Targeting autophagy enhances sorafenib lethality for hepatocellular carcinoma via ER stress-related apoptosis. Autophagy.

[32] Zhang Z., Zhou X., Shen H., Wang D., Wang Y. (2009). Phosphorylated ERK is a potential predictor of sensitivity to sorafenib when treating hepatocellular carcinoma: Evidence from an in vitro study. BMC Med..

[33] Oh S.J., Erb H.H., Hobisch A., Santer F.R., Culig Z. (2012). Sorafenib decreases proliferation and induces apoptosis of prostate cancer cells by inhibition of the androgen receptor and Akt signaling pathways. Endocr. Relat. Cancer.

[34] Haga Y., Kanda T., Nakamura M., Nakamoto S., Sasaki R., Takahashi K., Wu S., Yokosuka O. (2017). Overexpression of c-Jun contributes to sorafenib resistance in human hepatoma cell lines. PLoS ONE.

[35] Tai W.T., Shiau C.W., Chen H.L., Liu C.Y., Lin C.S., Cheng A.L., Chen P.J., Chen K.F. (2013). Mcl-1-dependent activation of Beclin 1 mediates autophagic cell death induced by sorafenib and SC-59 in hepatocellular carcinoma cells. Cell Death Dis..

[36] Sun C., Gao W., Liu J., Cheng H., Hao J. (2020). FGL1 regulates acquired resistance to Gefitinib by inhibiting apoptosis in non-small cell lung cancer. Respir Res..

